# Footprint morphology sheds light on running strategies in non-avian theropods

**DOI:** 10.1038/s41598-025-31361-y

**Published:** 2026-01-07

**Authors:** Ignacio Díaz-Martínez, Pablo Navarro-Lorbés, Erik Isasmendi, Adrián Páramo, Francesc Gascó-Lluna, Angélica Torices, Javier Ruiz, Luis Ignacio Viera, Patxi Sáez-Benito, James Farlow, Giuseppe Leonardi, Xabier Pereda-Suberbiola, Paolo Citton

**Affiliations:** 1 Departamento de Ciencias de la Tierra y Física de la Materia Condensada, Facultad de Ciencias, Universidad de Cantabria, Santander, 39005 Cantabria Spain; 2 Paleoymás S.L. Pol. Empresarium, Zaragoza, 50010 Spain; 3 Departamento de Ciencias de la Tierra, Aragosaurus-IUCA: Recursos Geológicos y Paleoambientes, Universidad de Zaragoza, Zaragoza, 50009 Spain; 4 Departamento de Geología/Geologia Saila, Facultad de Ciencia y Tecnología/Zientzia eta Teknologia Fakultatea, Universidad del País Vasco/Euskal Herriko Unibertsitatea (UPV/EHU), Leioa, 48940 Bizkaia Spain; 5 Scientific Computation Research Institute (SCRIUR), University of La Rioja, Logroño, 26004 Spain; 6 Centro de Interpretación Paleontológica de La Rioja, Igea, La Rioja, 26525 Spain; 7 Museo de Benagéber, Benagéber, 46173 Valencia Spain; 8 Departamento de Geodinámica, Estratigrafía y Paleontología, Universidad Complutense de Madrid, Madrid, 28040 Spain; 9 Department of Biological Sciences, Purdue University Fort Wayne, Fort Wayne, IN 46805 USA; 10 Istituto Cavanis, Dorsoduro 898, Venezia, 30123 Italy; 11 Instituto de Geociências-Geology Department, Universidade Federal do Rio de Janeiro, CCMN, Cidade Universitária, Ilha do Fundão, Rio de Janeiro, 21949-900 RJ Brazil; 12 Instituto de Investigación en Paleobiología y Geología (IIPG, CONICET-UNRN), Avenida Roca 1242, General Roca, 8332 Río Negro Province Argentina

**Keywords:** Dinosaur, Locomotion, Early Cretaceous, Cameros Basin, Spain, Ecology, Ecology, Evolution, Zoology

## Abstract

This study analyzes two trackways of the fastest running theropods in the fossil record, offering a rare opportunity to examine dinosaur biomechanics during high-speed locomotion. We focus on the distinct three-dimensional morphologies of footprints—La Torre 6B-01 and La Torre 6A-14 (Early Cretaceous, La Rioja, Spain)—produced by similar theropod trackmakers on the same surface. The La Torre 6B-01 footprints range from digitigrade to subdigitigrade, whereas the La Torre 6A-14 tracks vary from fully digitigrade to digitigrade with an elongated metatarsophalangeal area. These differences are interpreted as reflecting distinct phases of running locomotion, likely linked to changes in trackmaker behavior. Comparable morphologies occurred in other trackways that have been interpreted as produced by running trackmakers, from the Early Jurassic to the Late Cretaceous. These examples emphasize how different phases of a run—combined with variations in force exertion and autopodium positioning—can strongly influence footprint morphology. Our findings reveal that theropod dinosaurs employed running strategies more complex and dynamic than previously recognized. Subtle footprint features, such as the presence or absence of metatarsophalangeal impressions, can be directly tied to changes in posture, weight distribution, and muscular activity. This approach provides new perspectives on biomechanical evolution, showing that locomotor diversity and limb morphology shaped dinosaur adaptation and diversification.

**Supplementary Information:**

The online version contains supplementary material available at 10.1038/s41598-025-31361-y.

## Introduction

The three-dimensional morphology of a vertebrate track, prior to any post-formational modification, results from the complex dynamics between limbs and substrate during track formation^1^. Autopod anatomy and external morphology, including soft-tissues^2,3^, substrate properties^4,5^, limb dynamics^6,7^, and behavior^8^ are pivotal in defining the resulting morphologies of tracks. One of the most exciting challenges of vertebrate paleoichnology is to infer the possible condition of the aforementioned factors while footprints were formed, to get insights into ancient producers and paleoenvironments in which they dwelt and left their traces (see ^9,10^ and references therein). General axioms widely accepted in vertebrate ichnology, for instance (i) the same producer may leave different tracks; (ii) different producers may leave similar tracks, open a plethora of different questions, concerning under which conditions the same type of producer, trampling on the same substrate under the same type of behavior, would impress similar tracks^11^. One goal of ichnological analysis lies in the chance of deciphering whether or not the footprint morphology can be used to discriminate subcategories that exist among a single type of locomotion.

Trackways of running theropods are inferred when the measured stride length results three times greater than the trackmaker’s estimated acetabular height^12^. Trackways of this kind of locomotion are scarce in the worldwide dinosaurian ichnological record (^13^ and references therein). Commonly, the analysis of trackways impressed by running theropods relies on numerical calculation of speed, based on biomechanical formulas, and variations in measured pace angulation, pace and stride lengths (^14–22^, but see ^23^ for a critique of velocity calculations using fossil trackways). Recurrent ichnological features on single footprints making up a trackway and differences among resulting-track three-dimensional morphologies between different trackways of running theropods are seldom taken into consideration.

**Fig. 1:**
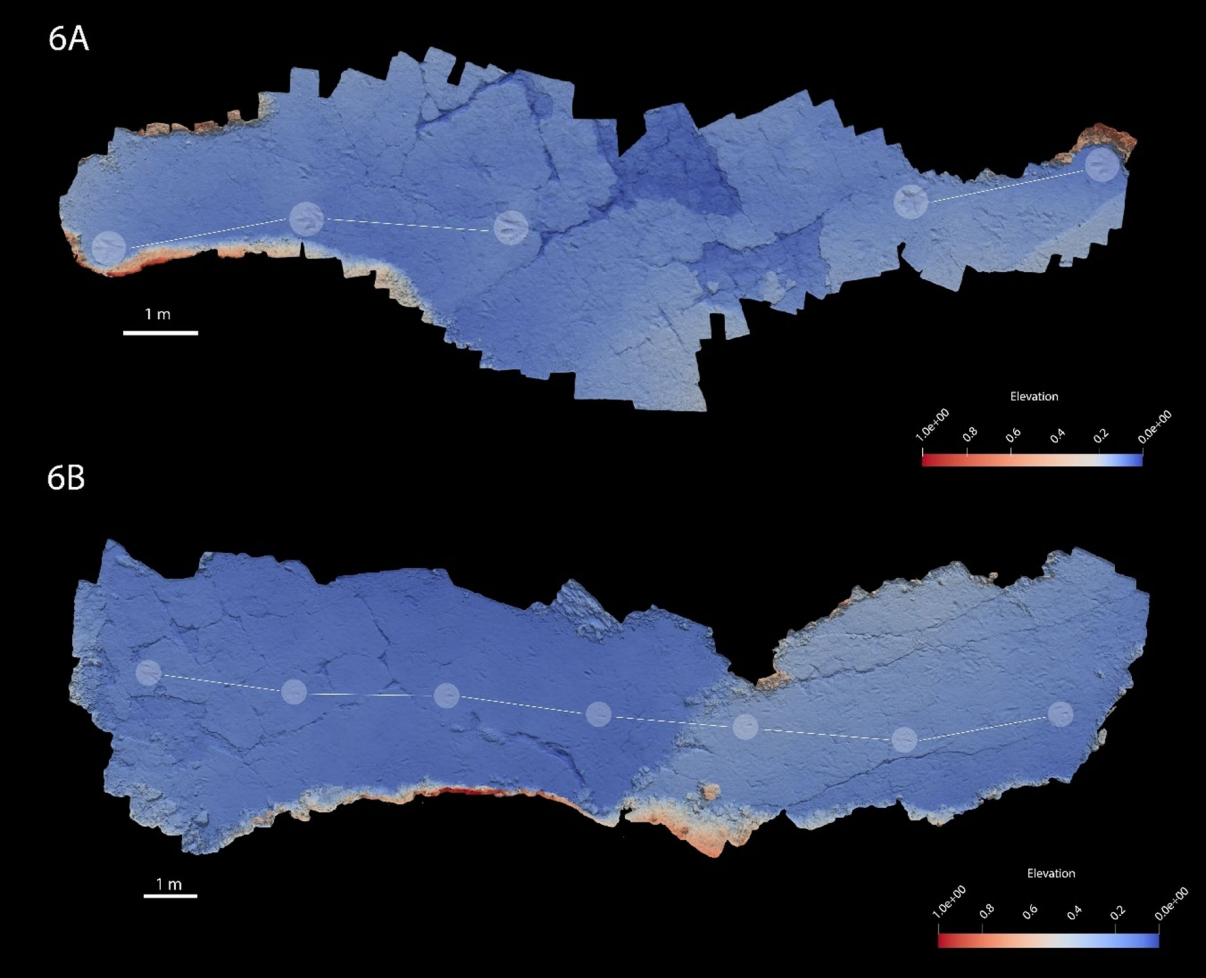
False-colour depth map of the (**a**) La Torre 6 A and (**b**) La Torre 6B based on 3D photogrammetric models. Footprints of the La Torre 6 A-14 and La Torre 6B-01 are highlighted in each image. The false-color depth map were generated with Paraview 5.11.1, a free software (https://www.paraview.org//).

The Lower Cretaceous La Torre 6 A-6B tracksites of La Rioja (Cameros Basin, Spain) preserve two trackways that represent evidence of some of the highest speeds ever calculated, reaching values between 30 and 40 km/h^13,24^ (Fig. 1). According to the authors, the trackways were made by the same type of producer running on the same tracking surface. Interestingly, tracks of each trackway, excluding internal variability, present different three-dimensional morphologies. Footprints belonging to La Torre 6B-01 trackway only consist of the distal portion of digits, while those belonging to La Torre 6 A-14 preserve more complete tracks, even with an elongated area consistent with the impression of metatarsophalangeal portion of producer autopods^13^. Therefore, if both trackways were produced by the same type of theropod moving with the same behavior in the same type of substrate, why do the tracks of these two coeval running trackways look so different?

To answer this question, we propose two possible reconstructions about the dynamics (i.e., the continuous interaction between autopods and the substrate where the tracks were formed) leading to observed track morphologies, for trackways La Torre 6 A-14 and La Torre 6B-1, respectively. Our proposal evaluates whether there is chance for discriminating different conditions within the same behavior by first considering footprint three-dimensional morphology, to improve quality of ichnological interpretations beyond simply measuring trackway parameters.

## Materials and methods

The La Torre 6 A-6B tracksites crop out at the locality of Igea (La Rioja, Spain). This site is geologically situated in the upper part of the Enciso Group (uppermost Barremian-lower Aptian and likely lowermost upper Aptian^25,26^) in mixed carbonate-siliciclastic lacustrine-palustrine deposits, and the studied trackways are preserved in situ as concave epireliefs at the top of the same limestone level (see ^13^ for a complete geographical and geological settings).

**Fig. 2:**
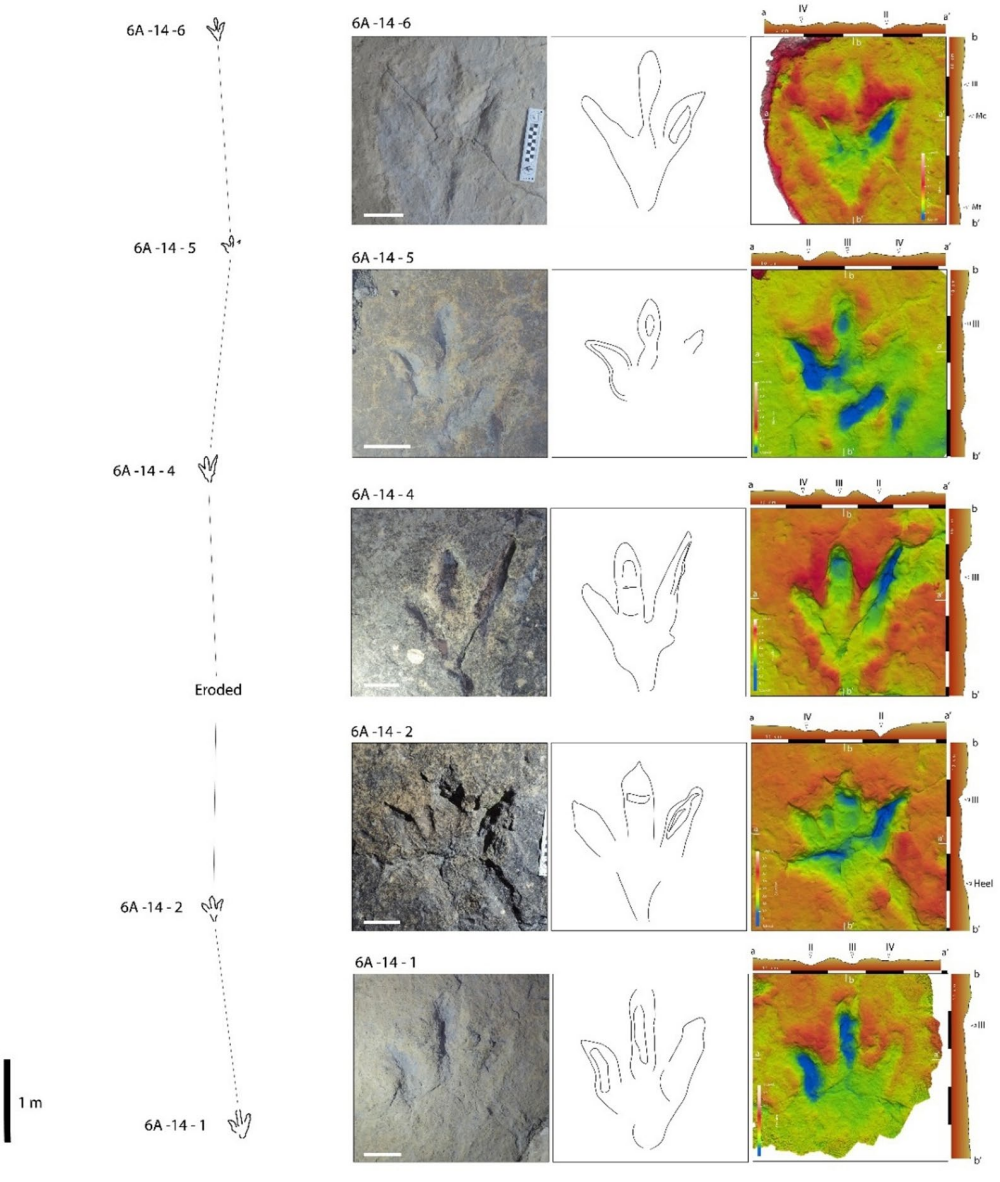
La Torre 6 A-14 trackway. Each footprint is represented by a photograph, interpretative sketch and false color image with cross-section profiles. Scale bar: 10 cm. The false-color depth map were generated with Paraview 5.11.1, a free software (https://www.paraview.org//).

**Fig. 3:**
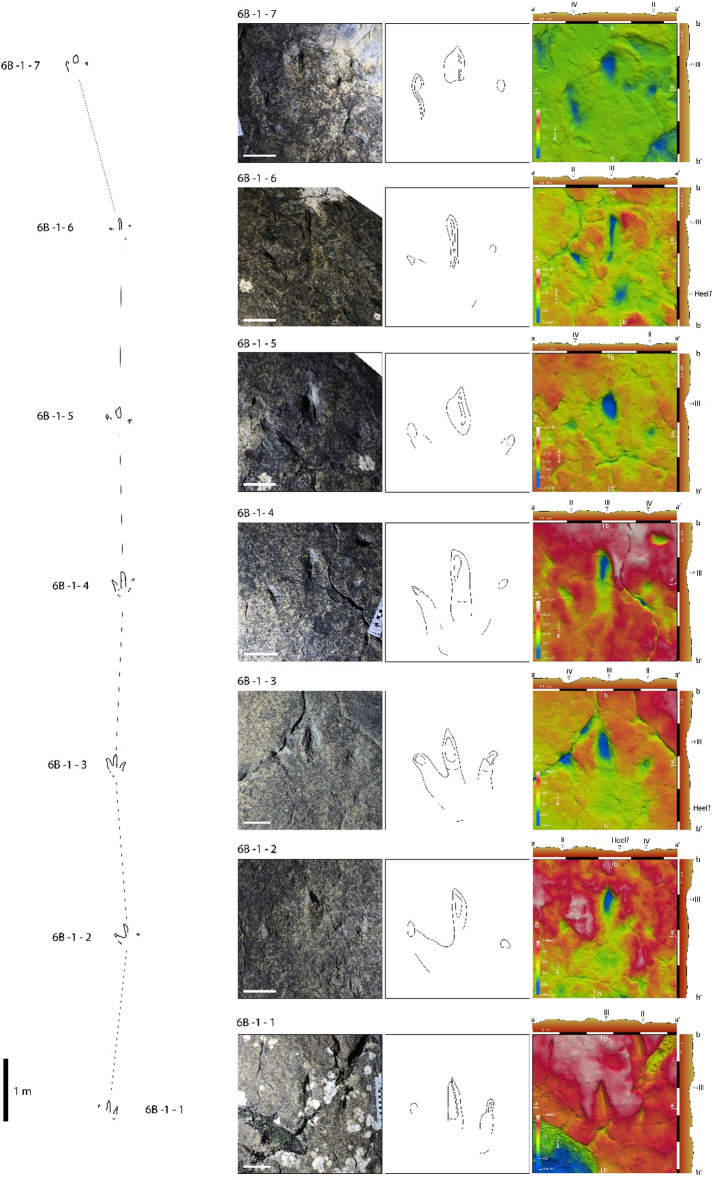
La Torre 6B-01 trackway. Each footprint is represented by a photograph, interpretative sketch and false color image with cross-section profiles. Scale bar: 10 cm. The false-color depth map were generated with Paraview 5.11.1, a free software (https://www.paraview.org//).

Our work is mainly based on detailed analysis of two running theropod trackways found at the tracksites La Torre 6 A and La Torre 6B. These trackways were previously described in other studies^13,23,27^. According to Agirrezabala et al.^27^, these two trackways are among 14 trackways at La Torre 6-A and 34 at La Torre 6-B. Both outcrops are in close proximity to each other but separated by vegetation and debris; however, field observations strongly suggest that they are equivalent stratigraphically^13^. The trackways, and their footprints, are designated according to the previous authors^13,26^. The La Torre 6 A-14 trackway preserves five footprints (La Torre 6 A-14-1, La Torre 6 A-14-2, La Torre 6 A-14-4, La Torre 6 A-14-5, and La Torre 6 A-14-6, with La Torre 6 A-14-3 lost by erosion) (Fig. 2). La Torre 6B-01 is a continuous trackway of seven footprints (La Torre 6B-01-1, La Torre 6B-01-2, La Torre 6B-01-3, La Torre 6B-01-4, La Torre 6B-01-5, La Torre 6B-01-6, La Torre 6B-01-7) (Fig. 3). The measurements of each footprint and trackway are extracted from Navarro-Lorbés et al.^13^ (see Supplementary Material Table S1 and S2).

To reconstruct the putative dynamics of track formation leading to the observed three-dimensional morphologies of the tracks, a qualitative analysis has been performed. The process of footprint formation involves multiple and continuous interactions between autopods and a compliant substrate^28–30^ during a cycle of locomotion, where functionally active portions of the autopods transfer loads from the limbs to the ground^31^. Thulborn and Wade^32^ described three main phases, the touch-down (T), the weight-bearing (W), and the kick-off (K) phases, to describe autopod-substrate interactions during a cycle of locomotion. The touch-down phase includes the forward and downward motion, and the initial contact of the autopod with the substrate; the weight-bearing phase indicates the full contact of the functional autopod with the substrate and involves the transition of the center of gravity of the producer over the autopod; the kick-off phase consists in the transfer of the body weight to the functional acropodium (i.e., digits) to accomplish the forward impulse of the body, and concludes with lifting off the ground and recovering of the autopod.

Our analysis primarily considers the footprint three-dimensional morphology including differential depth of impression (digits II and III which are the deepest parts of the footprint), to get information about gait mechanics, similarly to previous contributions dealing with detailed footprints of the Paleozoic and Mesozoic eras^33–37^. The material under study fulfills the requirements to conduct such analysis. Footprints were relatively shallow as observed during site excavation. Under these conditions differential depth of impression may be used, even if with great caution, as a proxy of pressure distributions during locomotion^38^.

Analysis of differential depth of impression relates also to the definition of possibly multiple conditions of axony^39^ in the observed tracks, adopting in this case its functional meaning more than the morphological and dimensional one^40^.

Data acquisition was conducted directly in the field and through the creation of photogrammetric three-dimensional representations^41^. These models were produced utilizing imagery captured by a Canon EOS 1200D and 80D equipped with an EF-S 18–55 mm II and Sigma 17–50 mm EX DC lenses, processed through Agisoft Metashape Professional software, version 1.6.1. Model generation employed two distinct approaches:^1^ constructing an orthomosaic of the entire trackway, and^2^ creating models of individual footprints. The trackway orthomosaic was derived from overhead images compiled into one single image, facilitating continuous measurement across the trackway (Figs. S1 and S2). Specifically, for the La Torre 6B trackway, 200 overhead images were utilized to construct the orthomosaic; similarly, for the La Torre 6 A trackway, 176 images were employed. In contrast, the individual footprint models were developed from multiple photographs captured from various angles and orientations, enabling the precise rendering of each footprint in three dimensions. This method allowed not only obtaining dimensional measurements but also analysis of the footprints’ detailed morphology. For each footprint, approximately 50 to 60 images were necessary to achieve a high-resolution model capable of capturing minute details and textures, particularly in regions of intricate geometry, with a resolution of 1.5 mm. 3D models are placed in the open repository Figshare as 10.6084/m9.figshare.29651600.

## Results and discussion

### Description of La Torre 6 A-14 and La Torre 6B-01 theropod running trackways

Trackway La Torre 6 A-14 is quite straight and measures 13.07 m in length. The unique measured pace angle equals 169°. Pace and stride lengths are quite constant, ranging from 250 to 270 cm (mean value 265 cm) and from 527 to 532 cm (mean value 529.5 cm), respectively. Footprint rotation is negative and equals − 3.8° (measured on footprint La Torre 6 A-14-5). All the footprints that constitute the trackway are slightly longer than wide. Mean footprint length equals 31.9 cm (measured without the elongated metatarsophalangeal area), whereas the mean footprint width is 30.2 cm. Total divarication angle, measured between digits II and IV, ranges from 44° to 60° (mean value 52).

Regarding the track three-dimensional morphology, the footprints of La Torre 6 A-14 are characterized by a posterior area most likely corresponding, from an anatomical standpoint, to the metatarsophalangeal portion, including soft-tissue, of the producer autopods (Fig. 2). In all the footprints except La Torre 6 A-14-5, this portion is proximally tapered, appearing somewhat elongated in La Torre 6 A-14-4, otherwise with a roughly rounded contour. Free digit traces are clearly impressed, and digit III is the longest. The digit II trace has a proximally rounded contour while distally, it bends medially (i.e., towards the midline) in La Torre 6 A-14-1, La Torre 6 A-14-2 and La Torre 6 A-14-5. It results almost straight in La Torre 6 A-14-4 and La Torre 6 A-14-6. In La Torre 6 A-14-2 and La Torre 6 A-14-4, digit II traces are also characterized by a narrow groove longitudinally extended in the central portion of the trace, which most likely resulted from the claw scratching the trampled surface. Digit III impressions are almost straight and display pointed and sharp tips, but not clear claw traces. Traces of digital pads in digit III are indicated by bulges and constrictions on the medial and lateral margins in La Torre 6 A-14-5, or by shallow rims transversally oriented with respect to digit main axis in La Torre 6 A-14-2 and La Torre 6 A-14-4. Digit IV traces have an oval to sub-triangular outline and distally display pointed and sharp tips which have the appearance of claw traces in La Torre 6 A-14-2, La Torre 6 A-14-4 and La Torre 6 A-14-5.

In general, the proximal portion of footprints corresponding to the metatarsophalangeal area is the shallowest impressed portion, appearing in some cases only faintly impressed. A similar depth distribution characterizes traces of digit IV, as can be observed from the digital elevation models obtained through photogrammetric 3D meshes. Traces of digit II are most deeply impressed portion in all the footprints (ranging 1.29 cm to 4.08 cm) and are characterized by a proximodistally homogeneous depth of impression, reaching paroxysm when scratch marks occur.

Finally, traces of digit III are characterized by a depth of impression that may be comparable to that observed on traces of digit II, for instance in footprint La Torre 6 A-14-1, but only in the proximal and centro-distal portions of the trace, the distal tip being less deeply impressed. In the other footprints, the trace of digit III is characterized by a lesser depth of impression, which can be comparable with that observed in traces of digit IV in the proximal and central portions. Within the digit III trace, the distal portion corresponding to the most distal phalanx behind the claw imprint is always the more deeply impressed.

The trackway La Torre 6B-01 measures 16.66 m in length and, like La Torre 6 A-14, is quite straight until the last footprint, whose orientation suggests a change of direction of the theropod towards the left, which is reflected in footprint morphology, with the penultimate footprint to the direction change being slightly different from the rest of trackway. Pace angulation ranges from 164°, measured in La Torre 6B-01-6, to 178° (mean value 172.2°). Pace and stride lengths are quite constant, ranging from 265 to 287 cm (mean value 279.6 cm), and from 543 to 569 cm (mean value 555 cm), respectively. The mean value of footprint rotation is positive (6.8˚) ranging from 3.3˚ to 11.8˚. Footprints are longer than wide. Mean footprint length equals 29.0 cm, while mean footprint width is 26.97 cm. The total divarication angle, measured between digits II and IV, presents a mean value of 55.96˚, with values ranging from 46.89˚ to 65.78˚. The highest and lowest values occur in the two last footprints, where a change in direction of movement is detected. If those values are excluded, the divarication angle ranges from 51.06˚ to 63.14˚, being the mean value 55.81˚. In each footprint, digit III is the longest.

The three-dimensional morphology of footprints is clearly different from that observed in footprints of trackway La Torre 6 A-14 (Fig. 3). In this case, the proximal portion corresponding to the anatomical metatarsophalangeal area is not impressed (except for a very faint impressed area on La Torre 6B-01-3), and all the footprints are only represented by traces of the central and distal portions of free digits. Traces of digits II and IV are characterized only by the impression of the distal portion of digits, corresponding to the tissue that covers the pre-ungual and ungual phalanges. In traces of digit II, this impression is oval to triangular in outline, in one case with a longitudinal groove posteriorly directed and most likely resulting from the claw scratching the sediment surface (La Torre 6B-01-3).

Only in footprints La Torre 6B-01-1, La Torre 6B-01-3 and La Torre 6B-01-4 can the more proximal portions of digits be observed as a faintly impressed elongated area. Traces of digit IV are even more faintly impressed than those of digits II and III, except for footprint La Torre 6B-01-7; in nearly all the footprints, the distal portion of digit IV is oval and pointed, but in footprint La Torre 6B-01-3 it is similar to the trace of digit II, and associated with a longitudinal groove. Traces of digit III are more completely impressed than traces of digits II and IV and characterized by a roughly elongated impression with acuminate claw traces. Almost all the digit III traces exhibit a longitudinal groove, running roughly parallel to the main axis of the digit (e.g., La Torre 6B-01-1, La Torre 6B-01-4, La Torre 6B-01-5 and La Torre 6B-01-6). This groove may run up to the central portion of digit trace or may extend over the entire length, in one case (e.g., La Torre 6B-01-6) terminating with a medial orientation, which is directed toward the trackway midline. In transverse cross-section, the digit III impression is asymmetric, with a sub-vertical wall in the medial sector and a lesser slope in the lateral surface. In almost all the tracks, there is a mediolateral rim in the middle area of the digit III imprint. The depth of impression is similar in the traces of digits II and IV; in some cases, the trace of digit II is slightly deeper. The traces of digit III are the most deeply impressed parts of the footprints, with depths ranging from 1.18 cm to 2.21 cm, showing a progressive increase from the proximal to the distal portion of the trace.

### Track formation dynamics

The three-dimensional morphology of a track results from a complex interaction between producer’s pedal anatomy and behavior, nature and conditions of the substrate^6,11^. Attempting to discriminate the contribution of each factor in determining ichnological features of resulting track morphology provides the chance to improve interpretations about the significance of dinosaur footprints.

As already noted, the two trackways record running theropods. The mean pace angulation in both trackways is similar, around 170°, while footprint rotation is negative in La Torre 6 A-14 (but only one datum is available) and positive in La Torre 6B-01. The relative strides (SL/h) calculated for La Torre 6 A-14 and La Torre 6B-01 (calculated with footprints 1, 3 and 6) are 3.98 and 4.81, respectively, while mean speed ranges from 6.5 to 10.3 m/s and from 8.8 to 12.4 m/s. According to the analysis conducted by Navarro-Lorbés et al.^13^, the dinosaur tracks of La Torre 6 A-14 and La Torre 6B-01 are true tracks^42^. Footprints constituting the two trackways clearly differ in the overall track morphology, if we consider the general outline and absolute dimensions, with footprints of La Torre 6 A-14 uniformly bigger than those of La Torre 6B-01. The absence of a clearly impressed area behind free digit traces in La Torre 6B-01 hinders a direct comparison with La Torre 6 A-14 in respect to this feature. Similarities between footprints of two trackways concern general morphology of digit traces, protrusion of digit III beyond II and IV and relative dimensions of free-digit area (i.e., FL/FW ratio measured in La Torre 6 A-14 without considering metatarsophalangeal area), equals to 1.06 and 1.07, respectively in better detailed footprints La Torre 6 A-14-1 and La Torre 6B-01-3. On these bases, Navarro-Lorbés et al.^13^ argued that the two trackways were impressed by distinct medium-sized individuals probably belonging to the same taxonomic group and proposed medium-sized theropods to be sought among early-branching tetanurans as most plausible producers, considering the Lower Cretaceous non-avian theropod record from the Iberian Peninsula.

Some of the footprints here discussed were excavated in the field by some of us and considering that no evidence of post-formational and pre-burial modifications were observed^42^, we consider that depth distribution can give clues about the original three-dimensional morphology. On the tracking surface, desiccation cracks occur locally over small areas with low density of dinosaur tracks. Footprints belonging to trackways La Torre 6 A-14 and La Torre 6B-01, similarly to other footprints on the tracking surface, are associated with low and narrow displacement rims. They appear slightly more developed in digit III impression, in medial zone in La Torre 6 A-14 and lateral zone in La Torre 6B-01, most likely as a result of the greater pressure applied to the ground by the producers’ acropodials during the weight and kick-off phases. Such configuration of displacement rims suggests that print emplacement occurred on hard and compact soil, with low moisture and high cohesiveness that would be related mainly to fluvial-lacustrine environments, like a floodplain or a lake shore^43^, the latter being in accordance with the paleoenvironmental reconstruction provided by Navarro-Lorbés et al.^13^. Local variations of moisture on the tracking surface may be inferred from the La Torre 6 A-14 trackway, which displays a slightly greater depth of impression than La Torre 6B-01, suggesting slightly higher sediment moisture at the time of passage. The difference in footprint depth between the La Torre 6 A-14 and La Torre 6B-01 trackways could indeed be partly explained by factors such as substrate consistency and trackmaker body mass. The sediment may have been softer when the La Torre 6 A-14 trackmaker passed, allowing deeper impressions, or the 6 A-14 trackmaker may have been slightly larger and heavier than that of La Torre 6B-01, resulting in greater penetration. However, the consistent differences in the location of maximum depth and claw longitudinal impressions — on digit II in La Torre 6 A-14 versus digit III in La Torre 6B-01 — as well as the distinct outline shapes beneath the digits, suggest that variations in foot posture or gait played the most important role. It is not, of course, being suggested that the sedimentary conditions prevailing at the time of track formation exerted no influence on the resulting three-dimensional morphology, as already noted above. Recurrent features observed in the individual footprints composing each of the examined trackways — which, despite the evident differences between them, remain consistent within and between both trackways — include, in particular: (i) the differential distribution of depths characterizing the impressions of the distal portions of the digits, which, as would be expected, represent the most deeply impressed elements; (ii) the negligible variation in the maximum depth recorded in the two trackways, which suggests only minimal differences in substrate conditions at the time of the two crossings; and (iii) the minimal differences in footprint depth when compared with other tracks preserved on the surface, which, irrespective of the gait recorded, point to an early-hardened substrate beneath the trampled surface. These features, which would likely be the first to be markedly affected by variations in the consistency of the trampled sediments or in substrate firmness, therefore provide stronger evidence for behavioural rather than substrate control in determining the resulting three-dimensional morphology.

Our reconstruction of the process of track formation from a dynamic perspective is based primarily on the three-phases model for cycle of locomotion discussed by Thulborn & Wade^32^. Based on the morphological and extra-morphological features observed in footprints of both trackways, position and orientation of producers’ autopods during running are summarized in Tables 1 and 2.

**Table 1:** Main features of La Torre 6 A-14 tracks during the footprint formation process.

Phase	Action	Autopod position	Features	Tracks
T	Contact	Low angle with respect to the substrate	Complete track with an elongated posterior area	6 A-14-2, 4, 6
W	Maximum weight	Metapodium moves forwardly Inward rotation	Pad impressions in digit III Claw impressions Developed rim in the medial zone of digit III Depth digit II impression Distal end of digit II inward rotated	6 A-14-2, 4, 5 6 A-14-2, 4, 5 6 A-14-1, 5, 6 6 A-14-1, 2, 4, 5, 6 6 A-14-1, 2, 5
K	Autopod elevation	Subvertical angle with respect to the substrate and continue the rotation	Digit II claw groove impression	6 A-14-2, 4

**Table 2:** Main features of La Torre 6B-1 tracks during the footprint formation process.

Phase	Action	Autopod position	Features	Tracks
T	Contact	Metapodium subvertical angle with respect to the substrate	Digit impressions Posterior area absent or very shallow	6B-01-1, 2, 3, 4, 5, 6, 7 6B-01-1, 2, 3, 4, 5, 6, 7
W	Maximum weight	Metapodium moves forwardly Outward translation	Claw impressions Depth digit impressions Small rim in the lateral zone of digit III	6B-01-1, 2, 3, 4, 5, 6, 7 6B-01-1, 2, 3, 4, 5, 6, 7 6B-01-1, 3, 4, 6
K	Autopod elevation	Metapodium low angle with respect to the substrate and continue the rotation	Rim in the middle zone of the digit III imprint Depth claw impressions Digit claw groove impressions Curved digit III claw groove impression	6B-01-1, 3, 4, 5, 6 6B-01-1, 2, 3, 4, 6 6B-01-1, 3, 4, 5, 6, 7 6B-01-1, 4, 5, 6, 7

**Fig. 4:**
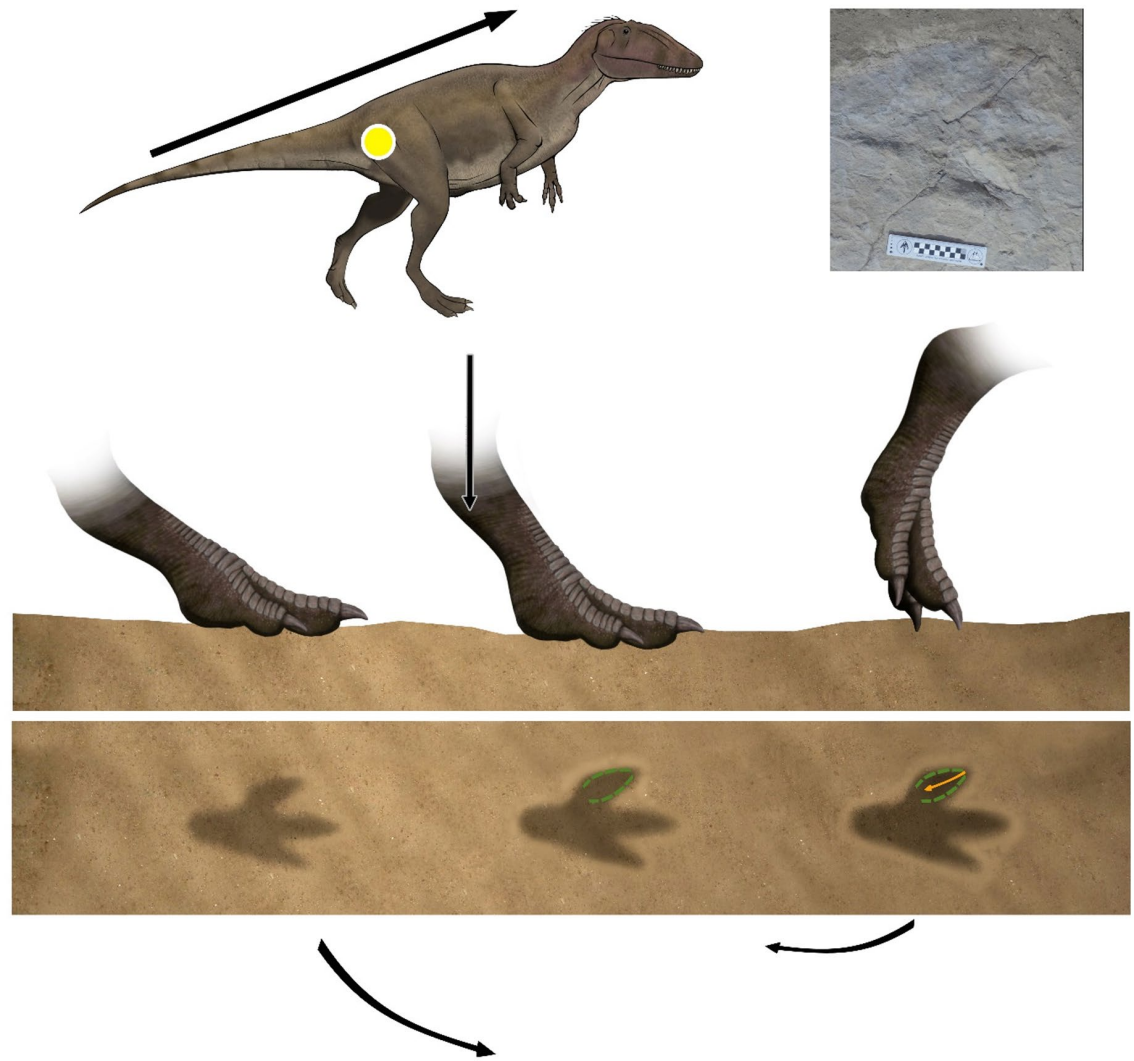
Hypothesized sequence of events leading to the formation of the La Torre 6 A-14 footprints, compared with track La Torre 6 A-14-6, illustrating successive foot movements and the resulting footprint morphology. The green dashed line corresponds to the W phase, while the yellow line represents the K phase. The art work was created by one of the authors (F. Gascó-Lluna).

During the touch-down phase the weight is shifted on the forward limb and the autopod makes little or no impression on the substrate^31,32^. The autopod of La Torre 6 A-14 trackmaker stepped with a low angle with respect to the substrate. In addition to the sole impression, typical of a digitigrade dynamic, the distal part of the metapodium is also preserved giving an elongated shape in the posterior area (Fig. 4). Probably, the trackmaker touched the ground with the functional autopodium (acropodium and part of metapodium) at the same time or before with the posterior area. On the other hand, the autopod of La Torre 6B-01 trackmaker probably touched the substrate with a subvertical angle. The distal part of the digits first contacts the substate and the metatarsophalangeal pad barely touches, if at all.

**Fig. 5:**
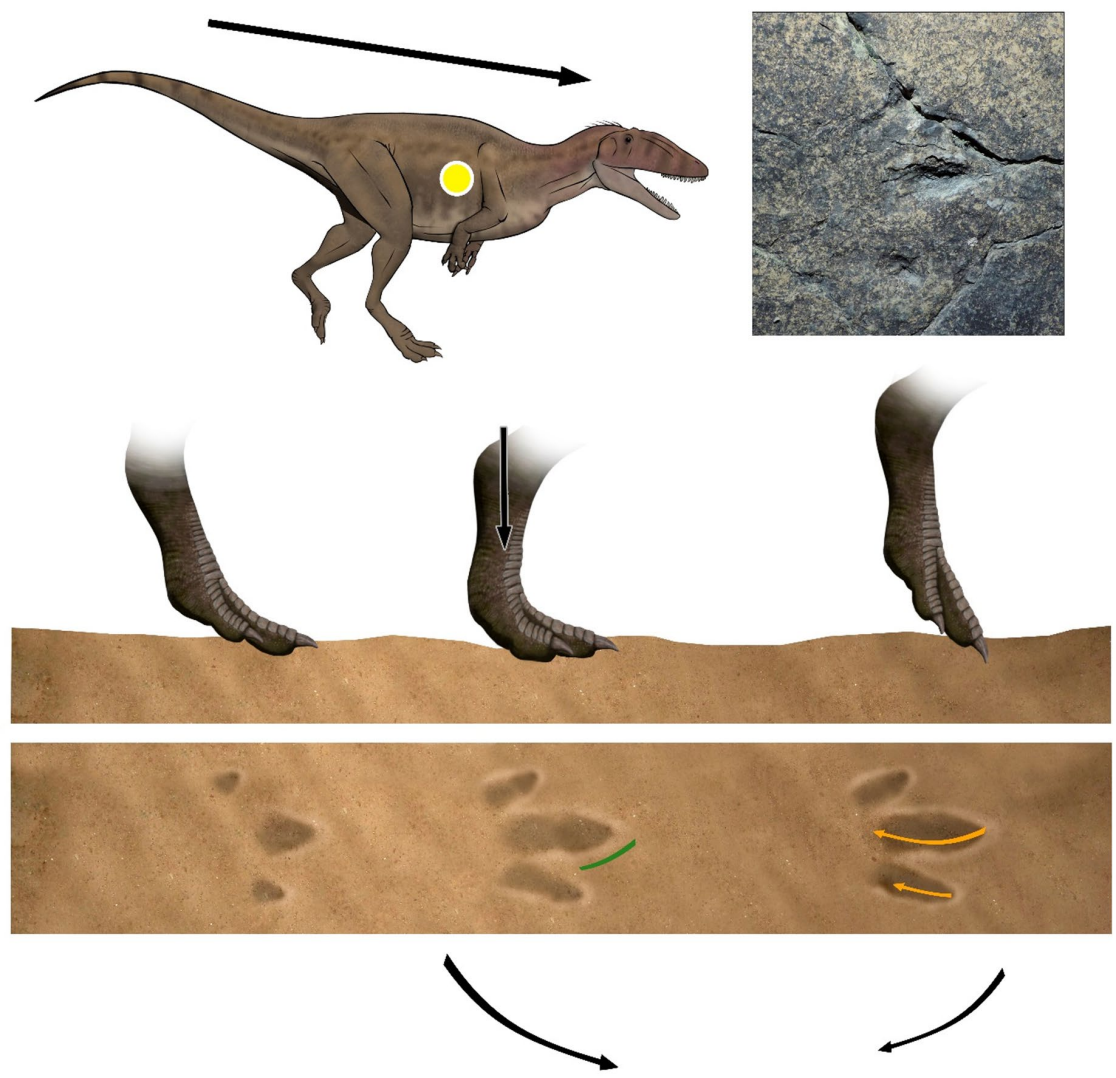
Hypothesized sequence of events leading to the formation of the La Torre 6B-01 footprints, compared with track La Torre 6 A-01-3, illustrating successive foot movements and the resulting footprint morphology. The green line corresponds to the W phase, while the yellow lines represent the K phase. The art work was created by one of the authors (F. Gascó-Lluna).

Thus, only the digits are well impressed whereas the posterior area is very shallow or absent, indicating a digitigrade to subdigitigrade gait (Fig. 5). In the weight-bearing phase the limb supports the body weight, the trackmaker’s center of gravity passes through the center of the autopod and the autopod sinks into the substrate^31,32^. In La Torre 6 A-14, the autopod exerts more force on the ground and the metapodium advances vertically. In this moment, the pad impressions in digit III and the claw traces are formed. Moreover, the trackmaker body center moved inward, loading the weight on digits II and III.

Consequently, the medial side of the autopod penetrated deeply into the ground (e.g., developed rim in the medial zone of digit III, depth digit II impression, distal end of digit II inwardly rotated). The metapodium of La Torre 6B-01 trackmaker advanced forwardly and the digits pressed hard on the substrate. Digits II and IV are mainly represented by the claw impressions, which are stuck in, resulting in one of the deepest parts of the track. An exception is the digit IV of La Torre 6B-01-7, which is elongated and the deepest one but coincides with a left turn in the trackway. On the other hand, the pressure passes along the digit III causing it to be preserved practically complete with a deeper anterior part and pointed claw impressions. The small rim found at the lateral area of the digit III impression would be produced during an outward translation.

Finally, during the kick-off phase the center of gravity passes ahead of the autopod which begins to lift off the ground starting with the metatarsophalangeal pad and ending with a backward sweep of the digits^31,32^. The trackmaker of La Torre 6 A-14 elevated the autopod of the substrate with the metapodium in a subvertical angle. This fact means that the distal part of the digits is not the deepest part of the print since the claws would not penetrate the substrate. As an exception, and because of inward rotation during W phase, a groove of the digit II claw is observed in some tracks. This digit would be the last to fully rise. The autopod of La Torre 6B-01 lifted off the ground with the metapodium forwardly rotated. First, the digits pulled back and began to lift. The claws deepened and a rim was formed in the middle zone of digit III. The claws imprinted longitudinal grooves parallel to the axis of each digit. Finally, the autopod moved inwardly and the longitudinal groove of digit III cut the posterior rim and is curved medially.

### Running trackways as a complex behavior

Terrestrial vertebrates employ a variety of locomotor modes when traversing the ground. For example, walking is typically used for slow-paced movement, whereas high-speed motion is achieved through running, trotting, galloping, or hopping^44^. Although the presence of an aerial phase is traditionally considered the defining characteristic of the transition from walking to running^45^, many animals can run slowly without an aerial phase by flexing the knees (i.e., “Groucho running”;^46,47^). This mode of locomotion has been observed in several bird species^48–50^ and has been hypothesized for theropod dinosaurs^50^. In contrast, an aerial phase is typically associated with fast running^44,48,51,52^, and was likely also adopted by the producers of the La Torre 6A and 6B trackways, given that these represent some of the fastest tracks discovered to date. Although it is uncertain whether the trackmakers were operating at the limits of their athletic capacity, they must have been running at high speeds to produce such trackways.

**Fig. 6:**
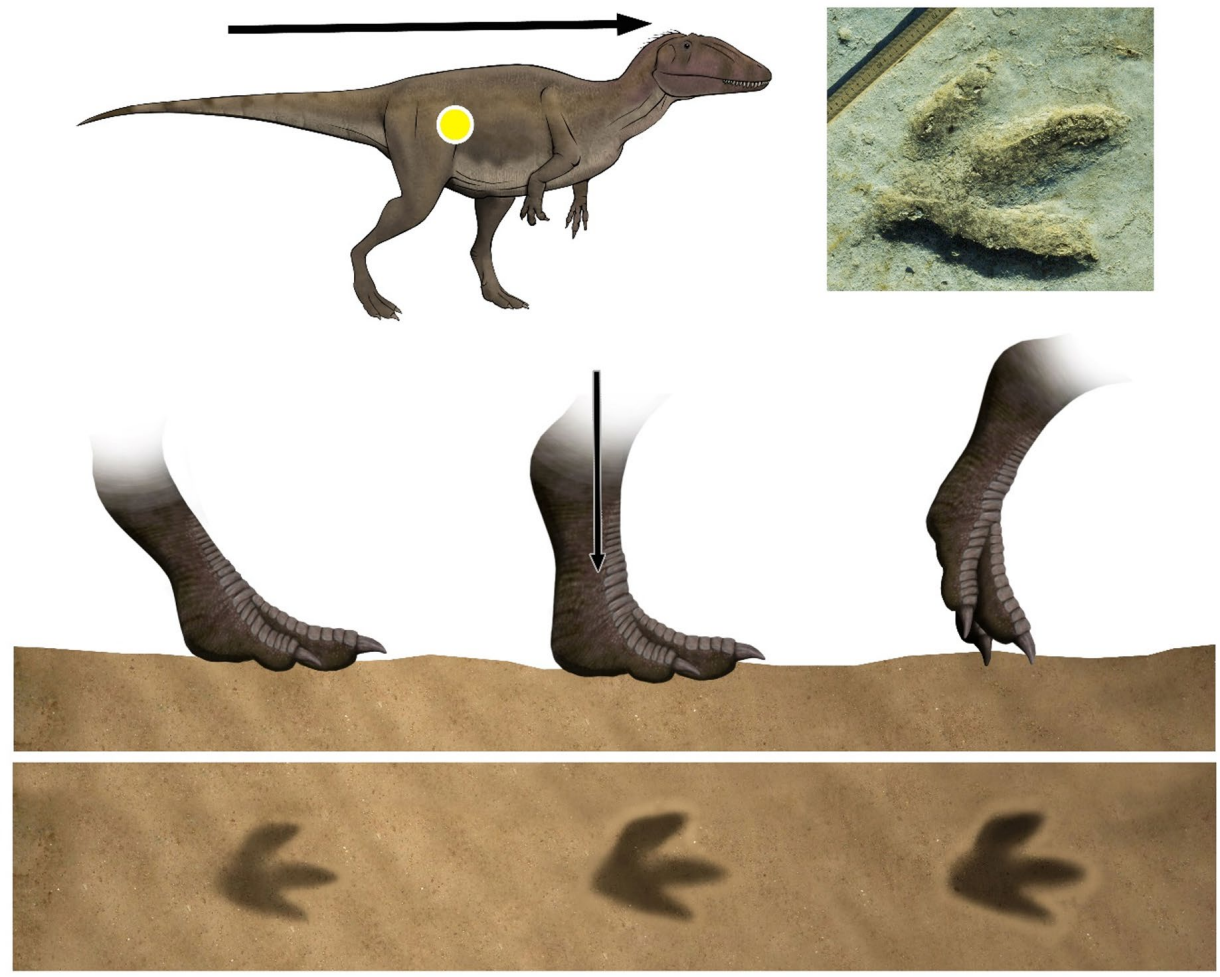
Hypothesized sequence of events leading to the formation of constant velocity-like running footprints, compared with track F6 A2 from Texas^15^, illustrating successive foot movements and the resulting footprint morphology. The art work was created by one of the authors (F. Gascó-Lluna).

Very fast running in bipedal dinosaurs may be analogous to sprint running in human athletes, which is generally divided into three phases: acceleration, constant velocity, and deceleration^53^. During these phases, variations occur in the relative forces exerted by the foot against the substrate, and these variations may be reflected in track morphology. In the acceleration phase, the center of mass is positioned more anteriorly, resulting in an increased anterior force during kick-off and producing tracks with more pronounced digit impressions^11,19,54^. Although such tracks are relatively rare in the theropod running record, they have been documented in an acceleration trackway from the Upper Cretaceous of Korea^19^, characterized by deeply impressed digit impressions and a very shallow or absent metatarsophalangeal region. Tracks of modern-day running emus have also been reported in which only the digits are impressed^55^. Both the emu tracks and those from La Torre 6B exhibit deeper impressions of digits III and IV, whereas the Korean trackway shows digit III as usually fully preserved—likely because it bears most of the weight—while digits II and IV are often only partially preserved. Notably, a trackway of three footprints from the Lower Cretaceous of Spain^56^ and a very fast trackway from the Lower Cretaceous of China^57^, composed of five footprints, display similar morphology, with deeper impressions at the tips of the digits and shallow or absent impressions at the metatarsophalangeal area. The morphology of the footprints from both China and La Torre 6B could be consistent with an acceleration trackway; however, in the former, stride lengths remain constant, whereas in La Torre 6B, variations in speed along the trackway, inferred from step lengths, suggest a more irregular gait. Therefore, the pronounced digit impressions may reflect higher exerted traction rather than acceleration per se.

During constant velocity, the center of mass is more similarly positioned relative to a walking gait, and although the load distribution patterns within the autopod may differ significantly^52,58^, based on experimental analyses with ostriches), the resulting tracks are generally similar, albeit deeper. For instance, the trackways from the two fastest theropod found in Lower Jurassic and Lower Cretaceous beds of the USA^15,21^, as well as the majority of running trackways (e.g.,^59^ from the Lower Cretaceous of Spain^16^; from the Lower Jurassic of the USA^60^; from the Upper Cretaceous of Korea^61^; from the Lower Cretaceous of Thailand, do not differ significantly from those of walking trackways, although^21^ noted that these tracks were deeper than other tracks preserved on the same bed (Fig. 6).

Finally, during the deceleration phase, the center of mass shifts posteriorly, increasing the force on the rear of the foot during initial contact and resulting in footprints with a more developed posterior region^11^. This characteristic is evident in three Lower Cretaceous running trackways from Brazil (SOPP3, SOPP4, and SOES1;^62^). La Torre 6 A-14 trackway shows a similar pattern (Fig. 4). Moreover, the digit II impression is the deepest in this trackway^13^ and is particularly well developed in SOES1. However, step length-based speed variations along the trackway^13^ do not support this interpretation, suggesting, if anything, a slightly increasing speed.

Based on the above exposed morphological data, it is possible to suggest that the studied trackways resulted by different behaviors during running. Considering the stance of producer’s autopods in dynamic condition (i.e., while moving) and following the approach based on pressure distributions and dynamic foot uses discussed by Michilsens et al.^63^ for mammals, it is also interesting to note a probable difference in dynamic stance between the studied trackways. Assuming a digitigrade stance in resting position for theropods, three-dimensional footprint morphologies would suggest three different conditions of digitiportality in the producers while running full in the case of La Torre 6 A-14 exerting greater pressure on digit II, digitiportal in constant velocity-like morphology with a pressure shared among the digits but greater in the III as in^15,21^, and tending to “sub-digitiportality”, pressing more on tips of digits III and IV, for La Torre 6B-01. These differences are likely related to the position of the foot and the center of mass during each tracking phase.

In running trackways, the Thulborn and Wade^32^ phases are temporally compressed and also shifted relative to those of a normal walking footprint. During the touch phases: (a) in the morphology of the La Torre 6A-14 trackway and similar footprints, the initial impression is made by the posterior surface of the metatarsophalangeal area, with comparatively low traction exerted; (b) in tracks with a constant velocity-like morphology, the metatarsophalangeal pad impression is clearly recorded; and (c) in higher-traction phases — such as in the La Torre 6B-01 type tracks — the impressions are made primarily by the digits. Footprint formation is completed during the kick-off phase, which is characterized by maximum pressure on the digits in the La Torre 6 A-10 track morphology, digit tip impressions in constant-velocity-like morphologies, and evidence of claw dragging, as observed in the La Torre 6B-01 trackway and similar footprints. These patterns have been documented in a limited number of running traces preserved in the fossil record, exclusively from theropods ranging from the Lower Jurassic to the Upper Cretaceous, thereby indicating that theropod running strategies were more complex than previously expected. This opens the door to linking subtle changes in footprint morphology—such as the presence or absence of metatarsophalangeal impressions—with variations in posture, weight distribution, and muscular behavior. Advances in computational biomechanics have demonstrated how variations in kinematic parameters (e.g., force distribution and center of mass positioning) directly influence locomotor performance^64^. These biomechanical insights highlight the relevance of muscular behavior and weight distribution in shaping footprint morphology. Furthermore, Bishop et al.^65^ emphasized the dynamic role of muscle-driven simulations in understanding the complex interactions between limb movement and substrate during locomotion. These studies collectively suggest that diversity in running strategies—evidenced by variations in computational simulations and the ichnological record—has played a key role in shaping both limb morphology and the adaptive diversity of dinosaurs. By extending this framework to fossil trackways, we propose that minor morphological changes in footprints can indicate underlying variations in posture and biomechanics, providing crucial insights into the evolution of dinosaur locomotion and their ecological diversification.

## Conclusions

This study provides new insights into the biomechanical complexity of theropod running by analyzing two of the fastest-known trackways preserved in the fossil record. The three-dimensional footprint morphology of the La Torre 6A-14 and La Torre 6B-01 trackways from the Lower Cretaceous of La Rioja (Spain) reveals distinct patterns. Our findings highlight that theropod running strategies were more sophisticated than previously recognized, involving different foot postures and load distributions depending on the phase of motion. This suggests that fossilized tracks can preserve detailed locomotor dynamics, offering a new avenue for reconstructing the biomechanics of extinct theropods beyond traditional speed estimates.

Furthermore, our study underscores the importance of considering three-dimensional footprint morphology in addition to trackway metric parameters. The presence or absence of metatarsophalangeal pad impressions, variations in digit depth, and claw drag marks reflect subtle yet critical differences in weight distribution, limb positioning, and muscular engagement. These findings align with recent biomechanical simulations, reinforcing the idea that locomotor diversity played a significant role in the adaptive radiation of theropods.

Ultimately, this research expands our understanding of dinosaur locomotion and highlights the potential of footprint analysis as a high-resolution tool for studying extinct vertebrate biomechanics. Future work integrating ichnological data with musculoskeletal modeling and track formation experiments will further refine our interpretations of theropod movement and evolutionary adaptations.

## Supplementary Information

Below is the link to the electronic supplementary material.

Supplementary Material 1

Supplementary Material 2

Supplementary Material 3

Supplementary Material 4

Supplementary Material 5

## Data Availability

The datasets generated and/or analysed during the current study are available in the Figshare [NAME] repository, 10.6084/m9.figshare.29651600.
