# Supplementary material for: Footprint morphology sheds light on running strategies in non-avian theropods
Source: Sci Rep. 2026 Jan 7;15:44217. doi: 10.1038/s41598-025-31361-y (PMC12780217; doi:10.1038/s41598-025-31361-y)
Supplement: Supplementary file 1 — Supplementary Material 1 [file 41598_2025_31361_MOESM1_ESM.docx]

| Footprint | L | W | Max D III | Max D II | PL | PA | Fr |
| --- | --- | --- | --- | --- | --- | --- | --- |
| 6B - 01 - 1 | 29.8 | 24.4 | 1.18 | 1.57 | 279 |  |  |
| 6b - 01 - 2 | 24.6* | 25.7 | 1.86 | 0.96 | 284 | 170 | 3.4 |
| 6B - 01 - 3 | 27.1 | 28.4 | 1.85 | 1.41 | 28 | 173 | 8.1 |
| 6B - 01 - 4 | 32* | 28.1 | 1.57 | 0.54 | 265 | 175 | 3.3 |
| 6B - 01 - 5 | 27.1* | 26.3 | 2.21 | 1.18 | 287 | 177 | 7.3 |
| 6B - 01 - 6 | 29.9 | 27.8 | 1.66 | 1.40 | 283 | 172 | 11.8 |
| 6B - 01 - 7 | 32.5* | 28.1 | 1.62 | 1.53 |  |  |  |
| Mean | 29.00 | 26.97 | 1.7 | 1.22 | 279 | 173 | 6.8 |

*Table S2. Measurements of trackway 6B- 01. L: Length; W: Width; Max D III: Maximum deepness of digit III impression; Max D II: Maximum deepness of digit II impression; PL: Pace Length; SA: Pace Angle; Fr: Footprint rotation. * Estimated measure. All measures in cm and degrees.*
