# Supplementary material for: Footprint morphology sheds light on running strategies in non-avian theropods
Source: Sci Rep. 2026 Jan 7;15:44217. doi: 10.1038/s41598-025-31361-y (PMC12780217; doi:10.1038/s41598-025-31361-y)
Supplement: Supplementary file 2 — Supplementary Material 2 [file 41598_2025_31361_MOESM2_ESM.docx]

| Footprint | L | W | Max D III | Max D II | PL | PA | Fr |
| --- | --- | --- | --- | --- | --- | --- | --- |
| 6A - 14 -01 | 35.2 | 31.1 | 1.77 | 1.96 | 259 |  |  |
| 6A - 14 -02 | 30.3 | 33.2 | 2.7 | 4.08 |  |  |  |
| 6A - 14 -03 |  |  |  |  |  |  |  |
| 6A - 14 -04 | 35.7 | 29.3 | 2.94 | fractures | 266 |  |  |
| 6A -14 -05 | 28.5* | 27.9 | 1.2 | 2.01 | 270 | 169 | -3.8 |
| 6A -14 -06 | 30.01 | 29.5 | 0.4 | 1.29 |  |  |  |
| Mean | 31.94 | 30.2 | 1.8 | 2.33 | 265 |  |  |

*Table S1. Measurements of trackway 6A- 14. L: Length; W: Width; Max D III: Maximum deepness of digit III impression; Max D II: Maximum deepness of digit II impression; PL: Pace Length; SA: Pace Angle; Fr: Footprint rotation. * Estimated measure. All measures in cm and degrees.*
