# Supplementary material for: Footprint morphology sheds light on running strategies in non-avian theropods
Source: Sci Rep. 2026 Jan 7;15:44217. doi: 10.1038/s41598-025-31361-y (PMC12780217; doi:10.1038/s41598-025-31361-y)
Supplement: Supplementary file 5 — Supplementary Material 5 [file 41598_2025_31361_MOESM5_ESM.docx]

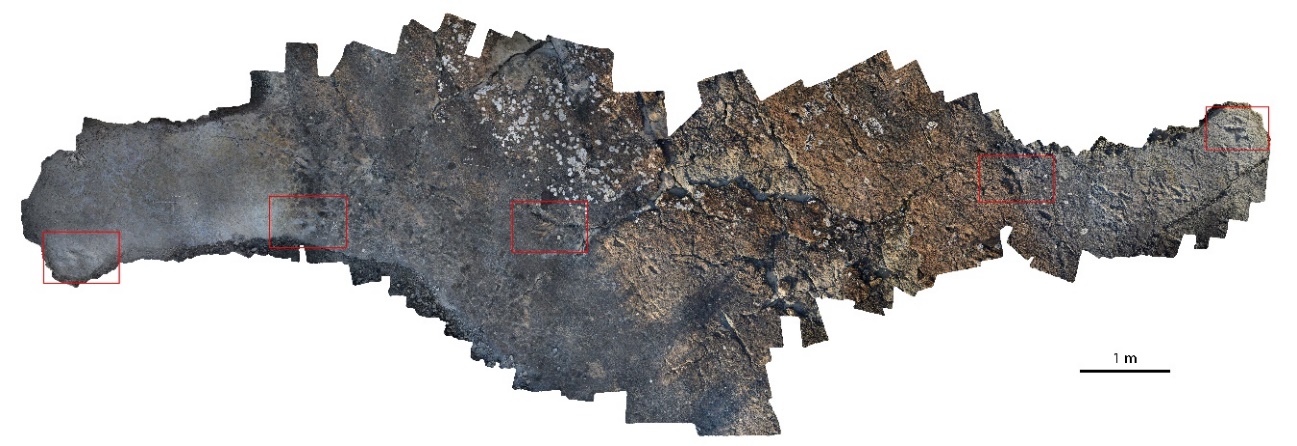


*Figure S1. Orthomosaic image of La Torre 6A. Footprints of the La Torre 6A-14 are shown within the red square. The orthomosaic was generated with Agisoft Metashape Professional 1.6.4 (https://www.* *https://www.agisoftmetashape.com//).*


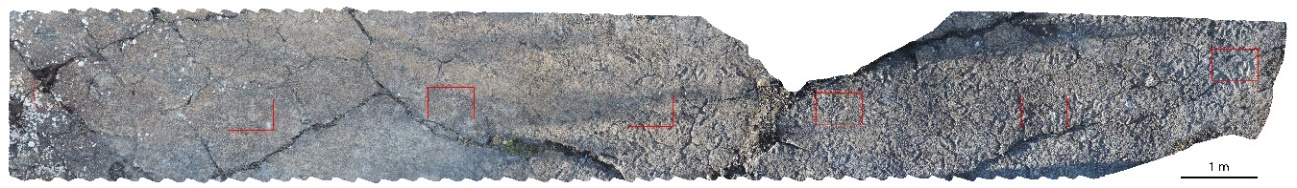


*Figure S2. Orthomosaic image of La Torre 6B. The footprints of La Torre 6B-01 are shown within the red square. The orthomosaic was generated with Agisoft Metashape Professional 1.6.4 (https://www.* *https://www.agisoftmetashape.com//).*
